# Non-Targeted Authentication Approach for Extra Virgin Olive Oil

**DOI:** 10.3390/foods9020221

**Published:** 2020-02-20

**Authors:** Didem Peren Aykas, Ayse Demet Karaman, Burcu Keser, Luis Rodriguez-Saona

**Affiliations:** 1 Department of Food Science and Technology, The Ohio State University, 100 Parker Food Science and Technology Building, 2015 Fyffe Road, Columbus, OH 43210, USA; aykas.1@osu.edu; 2 Department of Food Engineering, Faculty of Engineering, Adnan Menderes University, Aydin 09100, Turkey; 3 Department of Dairy Technology, Faculty of Agricultural Engineering, Adnan Menderes University, Aydin 09100, Turkey; demet.karaman@adu.edu.tr; 4 Kocarli Vocational School, Adnan Menderes University, Aydin 09100, Turkey; bkeser@adu.edu.tr

**Keywords:** authenticity, extra virgin olive oil, Raman, FT-IR

## Abstract

The aim of this study is to develop a non-targeted approach for the authentication of extra virgin olive oil (EVOO) using vibrational spectroscopy signatures combined with pattern recognition analysis. Olive oil samples (*n* = 151) were grouped as EVOO, virgin olive oil (VOO)/olive oil (OO), and EVOO adulterated with vegetable oils. Spectral data was collected using a compact benchtop Raman (1064 nm) and a portable ATR-IR (5-reflections) units. Oils were characterized by their fatty acid profile, free fatty acids (FFA), peroxide value (PV), pyropheophytins (PPP), and total polar compounds (TPC) through the official methods. The soft independent model of class analogy analysis using ATR-IR spectra showed excellent sensitivity (100%) and specificity (89%) for detection of EVOO. Both techniques identified EVOO adulteration with vegetable oils, but Raman showed limited resolution detecting VOO/OO tampering. Partial least squares regression models showed excellent correlation (Rval ≥ 0.92) with reference tests and standard errors of prediction that would allow for quality control applications.

## 1. Introduction

Counterfeiters target high-value products, including those with a strong brand name, deceiving consumers by substituting a high-value product with a less expensive or lower quality alternative. Although most food fraud concerns do not result in a public health or food safety crisis, these acts can lead to severe health hazards, as evidenced by oil fraudulently sold as olive oil that caused an outbreak of a condition known as the toxic oil syndrome, affecting 20,000 people, of which more than 300 died in Spain (1981) due to the ingestion of a food-grade rapeseed oil containing aniline derivatives sold for human consumption by street vendors [1]. To prevent olive oil adulteration, global governmental agencies (e.g., European Commission, United States Department of Agriculture, International Olive Council, Codex Alimentarius, German/Australian Standard, North American Olive Oil Association) have developed different standards to regulate olive oil by establishing a set of physical, chemical, and organoleptic characteristics [2]. A 2013 report by the U.S. International Trade Commission (USITC) indicated that current standards for extra virgin olive oil (EVOO) are widely unenforced leading to adulterated and mislabeled products in the market [3]. Common adulterants of EVOO include lower quality olive oils (refined, pomace, or lampante) or seed oils [4].

Numerous analytical techniques have been proposed to detect and control olive oil adulteration, including Ultraviolet-visible (UV–vis) absorption [5,6], front-face total fluorescence spectroscopy [7], vibrational spectroscopy [8,9,10,11], mass spectrometry [12,13,14], nuclear magnetic resonance [15,16,17,18,19,20], and techniques such as DNA-based methods [21] and electronic noses [22]. Most methods to detect olive oil adulteration have focused on targeted approaches, providing great selectivity and sensitivity for identification and quantification of pre-defined compounds or classes of compounds, but fail to detect emerging risks from unexpected adulterants [23]. On the other hand, non-targeted screening, which is currently at the heart of metabolomics, focuses on the detection of all compounds in a sample without any prior knowledge of chemical entities which can then be compared with the fingerprint profile of pure reference sample [24].

Advancements in semiconductors have allowed miniaturization and cost reduction of spectrometer components, leading to commercially available portable, handheld, compact, and micro-devices in the industry. Key enabling technologies leading to miniaturized structures have been fostered by developments in Micro Electro Mechanical Systems (MEMS), thin-film filters, solid-state lasers, light-emitting devices (LEDs) and alternative light sources, fiber optic assemblies, and high-performance detector arrays [25]. These devices have been at the forefront of cutting-edge technologies and have become progressively smaller and easier to use. Miniaturized devices can be taken to or placed at/in/on-line points of vulnerability along with complex food supply networks and moved from the confines of the relatively stable and controlled laboratory environment into the potentially more challenging and dynamic environs of the food supply chain (point-and-shoot) [26].

Limited information is reported in the literature regarding the detection of olive oil adulteration using non-targeted classification approaches. Mossoba et al. (2017) evaluated FT-NIR in conjunction with a partial least square analysis to predict EVOO authenticity of 93 samples collected from online and local grocery stores [27]. The authors developed an FT-NIR index based on two carbonyl overtone (5280 cm^−1^ and 5180 cm^−1^) absorptions and generated partial least squares regression (PLSR) models for four specific oils (refined, high oleic, high linoleic, and palm olein) based on the different fatty acid composition of the potential adulterants in EVOO [27]. FT-IR equipped with an attenuated total reflectance (ATR) accessory and combined with supervised pattern recognition techniques (soft independent modeling of class analogy (SIMCA) and partial least squares discriminant analysis (PLS-DA) have detected adulteration of EVOO with vegetable oils at levels above 10% [28,29]. Jimenez-Carvelo and others (2017) evaluated the use of FTIR-ATR and Raman spectroscopy (785 nm excitation laser) with different chemometric classification methods to detect adulteration of olive oil in blends with vegetable oils [30]. They successfully discriminated olive oils from blends containing over 10% vegetable oils by using PLS-DA and support vector machine-classification (SVM-C) for FT-IR and Raman analysis, respectively. Georgouli and others (2017) assessed the capabilities of a compact FTIR-ATR and a bench-top 1064 nm Raman spectrometers on the detection of EVOO adulteration with hazelnut oil (1–90%) mixtures by using a novel continuous locality preserving projections (CLPP) technique accompanied by a k-nearest neighbors (kNN) algorithm, reporting a classification rate ≥69% [31]. Although these studies have shown the capabilities of vibrational spectroscopy to detect EVOO adulteration with vegetable oils, they have not included lower quality olive oil (refined, lampante, or pomace), and most have been developed using a limited number of olive oil samples coming from restricted varietal origins and geographical areas, which limits their use as global methods to detect adulteration of olive oil (independently of the cultivars) with any edible vegetable oil [2,30].

This study aimed to develop an authentication program for EVOO using vibrational spectroscopy signatures combined with pattern recognition analysis for non-targeted screening of commercial EVOO samples and to generate prediction models for monitoring olive oil quality parameters.

## 2. Materials and Methods

A total of 151 olive oil samples were used in this study. Samples from Turkey (*n* = 91) were obtained from Aydin Commodity Exchange Laboratories in Aydin, Turkey, which monitors EVOOs for exportation to different countries. In addition, we included EVOO samples that were kindly provided by the California Olive Oil Council (*n* = 20) and samples purchased from grocery stores that included origins from Italy, Spain, Greece, Turkey, Tunisia, Portugal, and Peru (*n* = 40). Oils were placed in amber glass vials and stored at −18 °C until further analysis to minimize oxidation and any compositional changes.

### 2.1. Reference Methods

The fatty acid profile was determined using a fatty acid methyl ester (FAME) procedure. Fatty acid esterification was achieved by dissolving 100 μL olive oil sample with 10 mL of hexane in a glass tube, after which 100 μL 2N potassium hydroxide in methanol was added and the mixture was vortexed. An aliquot (1.5 mL) was placed into a microcentrifuge tube and rotated at 13.2 rpm for 5 min, and the solution was transferred into a borosilicate glass vial and stored at −18 °C until further Gas Chromatography (GC) analysis. FAMEs were analyzed using an Agilent 6890 series (Santa Clara, CA, USA) GC, equipped with a flame ionization detector (FID) and an HP G1513A autosampler and a tray. Fatty acids’ separation was achieved using HP-88 60 m × 0.25 mm × 0.2 µm column (Agilent 112-8867), and helium was used as a carrier gas. The injection volume was 1 µL with a split ratio of 20:1. The oven conditions were 110 °C for 1 min, to 220 °C (5 °C/min) hold for 15 min. The injector temperature was 220 °C, and the detector temperature was 250 °C. Fatty acids were identified by comparing each peak’s retention times against reference standards (Supelco^®^ 37 Component FAME Mix, Sigma Aldrich, St. Louis, MO, USA). GC analyses were carried out in duplicate.

### 2.2. Monitoring EVOO Quality Indices

Olive oil samples were analyzed for peroxide value (PV), free fatty acid (FFA) value, pyropheophytins (PPP), and total polar compound (TPC) tests. PV and FFA of the samples were determined using a Metrohm, 916 Ti-Touch (Herisau, Switzerland) automatic titrator. The PV test was performed using a Metrohm Pt Titrode electrode (Herisau, Switzerland), by following the AOCS official method Cd 8-53 [32] and expressed as meqO_2_/kg of oil. The FFA test was carried out using a Metrohm Solvotrode electrode (Herisau, Switzerland) and following the European Pharmacopoeia 5.0 01/2005:20501 modifications to the AOCS official method Ca 5a-40 [33]. FFA results were expressed in terms of the percentage of oleic acid. Pyropheophytin analysis was carried out by following the ISO 29841:2009/AMD 1:2016 [34] official method and by using a high-performance liquid chromatography (HPLC) (1100 Series, Agilent Technologies, Santa Clara, CA, USA) that was equipped with a G1311A quaternary pump, a G1322A degasser, a G1313 ALS autosampler, and a G1315B DAD detector (Agilent Technologies, Santa Clara, CA, USA). The separated pheophytin components were monitored at 410 nm. The results were expressed as relative proportions (%) of the analytes (pheophytin a and a’, and pyropheophytin a). Total polar compound (TPC) content was determined using Testo 270 oil tester (West Chester, PA, USA), according to the manufacturer’s operation guide and expressed as a percentage. All the reference tests were carried out in duplicate.

### 2.3. Vibrational Spectroscopy

Before the data collection, all the olive oil samples were heated to 65 °C in a lab oven (Precision Standard Incubator, PR205125G, Thermo Fisher Scientific, Waltham, MA, USA) to liquefy all the samples to the same level. FT-IR Spectroscopy: Spectra of each oil sample were acquired using a portable 5500a series compact Fourier-Transform IR spectrometer (Agilent Technologies Inc., Santa Clara, CA, USA) equipped with a temperature controlled, 5-reflections ZnSe crystal attenuated total reflectance (ATR) accessory, which was set to 65 °C to prevent fat solidification during the spectral collection. Thermoelectrically-cooled deuterated triglycine sulfate (dTGS) detector was used to measure the amount of light absorbed by the sample. Data collection was done in duplicate. A 75 μL oil aliquot was deposited onto the heated crystal. Spectra were collected over a range of 4000–700 cm^−1^ at 4 cm^−1^ resolution and by co-adding 64 scans, to improve the signal-to-noise ratio. Spectral data were displayed in terms of absorbance and viewed using Resolutions Pro Software (Agilent, Santa Clara, CA, USA). Raman Spectroscopy: Olive oil samples were heated (65 °C) in a lab oven before the analysis. Three milliliters of olive oil sample was placed in a quartz cuvette (Hellma Analytics, Mullheim, Germany) with the 10-mm light path for Raman analysis using a WP 1064 compact benchtop Raman spectrometer (Wasatch Photonics, Durham, NC, USA). The Raman spectroscopy was equipped with an Indium Gallium Arsenide (InGaAs) detector and a laser source operating at 1064 nm. The Raman spectra were collected from 250 to 1850 cm^−1^ with a resolution of 4 cm^−1^ and 3 scans were co-added and averaged to improve the signal-to-noise ratio of the spectrum with an integration time of 3000 ms. Between each sample, the background spectrum was acquired to eliminate environmental variations. Spectral data were displayed in terms of scattered light by the sample and viewed using Enlighten^TM^ software (Wasatch Photonics, Durham, NC, USA). Spectral data collection was done in duplicate.

### 2.4. Multivariate Data Analysis

The spectral data were imported as GRAMS (.spc) and Excel (.xls) files and analyzed using Pirouette^®^ multivariate statistical analysis software (version 4.5, Infometrix Inc., Bothell, WA, USA). FT-IR spectral data were transformed by smoothing (35 points) and taking the Savitsky–Golay second derivative (35 points with second order polynomial filter). Raman spectral data were preprocessed using mean-center and transformed taking the Savitsky–Golay second derivative (35 points with second order polynomial filter). Samples with high residual and leverage were re-evaluated and excluded if needed. The remaining samples were randomly divided into two sub-groups as calibration (80% of the total sample size) and validation (remaining 20%) sets.

Classification analyses of olive oils were performed by using soft independent modeling of class analogy (SIMCA), a supervised pattern recognition classification technique that uses previous knowledge about the category membership of samples to classify new unknown samples in one of the known classes based on its pattern of measurements [35]. The optimal number of principal components (PCs) for each class in the training set was determined by cross-validation, thus, lessening the effect of noise-laden PCs in the class model [35]. Class boundaries surrounding each class in the multivariate space represented the mean residual standard deviation of the training samples for a given class based on an F-statistic value set at a 95% specific confident interval. Interclass distances measure class separation in the multivariate space and interclass distances between groups of objects above 3.0 is regarded as significant to identify 2 groups of samples as different classes [36]. Lastly, the prediction of class membership was achieved by comparing the residual variance of an unknown to the average residual variance of the classes in the model using an F-test [37]. SIMCA only assigns unknown samples to the class for which it has the smallest residual, not forcing class assignments if the residual variance of an unknown exceeds the upper limit for every modeled class in the dataset. The sample will not be assigned to a class because it is either an outlier or comes from a class not represented in the model [37].

Partial least squares regression (PLSR) models were developed using infrared and Raman spectra and reference values obtained for fatty acid composition, free fatty acids, peroxide value, pyropheophytins, and total polar compounds. Separate PLSR models were developed for the infrared and Raman systems for each of the compounds of interest. PLSR combines features from principal component analysis (PCA) and multiple regression to solve problems involving high collinearity and to determine a set of dependent variables from a (very) large set of independent variables or predictors [38,39]. The PLSR algorithm extracts a set of orthogonal factors called “latent variables” that explains most of the variance from the X (spectra) and Y (concentration), generating an algorithm that diminishes the potential impact of large, irrelevant variations in the X matrix [39]. Leave-one-out cross-validation was applied to determine the optimal number of factors to prevent over- or under-fitting and to improve the modeling performance and the quality of the prediction [38]. The quality of the final model was evaluated based on the number of latent variables, loading vectors, standard error of cross-validation (SECV), the coefficient of determination (R-value), standard error of prediction (SEP), and outlier diagnostics, while outliers were determined using residual and Mahalanobis distances. The performances of models were determined by calculating the specificity and sensitivity based on true positive (TP, predicted result and actual label are both positive), false positive (FP, predicted result is positive while the actual label is negative), true negative (TN, predicted result and the actual label are both negative) and false negative (FN, predicted result is negative while the actual label is positive) classifiers [40].

## 3. Results and Discussion

### 3.1. Characterization of Olive Oils Using International Olive Oil Trade Standards

Olive oils were grouped as extra virgin olive oil (EVOO) (*n* = 77), virgin olive oil (VOO)/olive oil (OO) (*n* = 27), and adulterated olive oil with vegetable oils (corn, sunflower, soybean, and canola oil) (*n* = 47) according to information provided by the Aydin Commodity Exchange Laboratories (Aydin, Turkey) and California Olive Oil Council. Table 1 summarizes the information on reference analysis with regard to the levels of major fatty acids, free fatty acids (FFA), peroxide value (PV), pyropheophytins (PPP), and total polar compounds (TPC).

Fatty acid (FA) composition of the EVOO group (Table 1) showed that the five major FAs (16:0, 18:0, 18:1*n*-9, 18:2*n*-6 and 18:3*n*-3) fell within specified ranges set by the United States standards for grades of olive oil [41] and International Olive Council [42]. EVOO variation in FA levels among samples can be related to differences in geographic origin, variety, stage of maturity of the fruit, latitude, climatic conditions, storage, and extraction process of samples [43,44,45]. EVOO and VOO showed similar fatty acid profiles, except for a sample obtained from Peru that showed higher palmitic (18.1%) and linoleic (17.7%) but lower oleic (57.7%) compared to other VOO samples. On the contrary, adulterated olive oils with vegetable oils showed marked variation in FA composition (Table 1). For instance, olive oil adulterated with canola oil had lower palmitic acid (5.7%), while linoleic (28.5%) and linolenic (4.4%) acids were higher than pure olive oil. Adulteration of EVOO with corn oil resulted in a decrease in the levels of oleic acid (29.9%) and an increase in linoleic acid (58.6%) content.

The average FFA content of the EVOO and VOO/OO samples ranged from 0.4 ± 0.2% and 0.5 ± 0.5%, respectively. The main difference between EVOO and VOO resulted from their FFA content. According to the trade standards of the International Olive Council (IOC) (2018), the FFA content of EVOO, VOO, and OO cannot exceed 0.8%, 2.0%, and 1.0%, respectively [42]. FFA levels of adulterated EVOO samples with other vegetable oils ranged from 0.1% to 10.3% (2.1 ± 2.7%). In particular, two adulterated EVOO samples showed FFA levels of 9.0% and 10.3% that could be related to mixing olive oils with crude vegetable oil or waste cooking or frying oil. There is no FFA limit for the crude vegetable oils, van Doosselaere (2013) reported that crude palm oil FFA levels could reach levels of 20–25% because of the lipolytic enzymes of the fruit that were not handled properly [46]. The frying or cooking process increases the FFA content of vegetable oils since oils that contain high levels of polyunsaturated fatty acids are highly susceptible to hydrolysis, oxidation, and polymerization under a frying environment [47].

Peroxide value of olive oil samples were 9.8 ± 2.0, and 10.0 ± 2.5 meqO_2_/kg for EVOO and VOO/OO samples, respectively. According to the European Union Commission Regulations (EEC/2568/91), the PV limit for EVOO and VOO are 20 meqO_2_/kg, whereas the limit for OO is 15 meqO_2_/kg [48], and our findings were under the established limits for different grades of olive oils. Similar values for PV of EVOO and VOO, ranging from 6.2 to 11 meqO_2_/kg, were reported by Casal and others (2010) [49]. A high PV indicates that olives or paste were likely mishandled [50]. Adulterated olive oils with other vegetable oils showed PV ranging from 2.5 to 32.7 meqO_2_/kg, indicating that counterfeiters employ a wide array of oil quality, including freshly deodorized to highly oxidized vegetable oils.

Pyropheophytin (PPP) values of the samples were 11.5 ± 2.3, 13.2 ± 3.0, 19.8 ± 3.0% for EVOO, VOO/OO blends, and olive oil mixtures with vegetable oil samples, respectively. The PPPs are the breakdown products of chlorophyll in olive oil. The chlorophyll pigment initially breaks down to pheophytin (a and a’), and then into pyropheophytins, due to the decarbomethoxylation of chlorophyll and pheophytins, upon the effect of heat [51]. The elevated level of PPP indicates that the samples were oxidized and/or adulterated with cheaper refined oils and the limit of the total PPP should be lower than 15% in EVOO [52].

Average total polar compounds (TPC) of the EVOO, VOO/OO, and adulterated olive oils ranged from 5.2 ± 1.1%, 6.6 ± 1.5%, and 8.7 ± 2.4%, respectively. The TPC measures the polar fraction in oils that are composed of polymers (dimers, trimers, and highly polymerized compounds) and decomposition products (mono and diacylglycerols, FFAs, volatile compounds, cyclic, and non-cyclic monomers) [53]. The TPC limit for frying oil is 25% according to international legislation, and if an oil exceeds this limit it becomes unsuitable for human consumption [53].

Overall, the chemical quality parameters of EVOO and OO showed strong overlapping within minimum and maximum limits, making it challenging to use these parameters as reliable markers to identify potential adulteration to consumers.

### 3.2. Spectral Analysis of Olive Oil Samples

**Figure 1:**
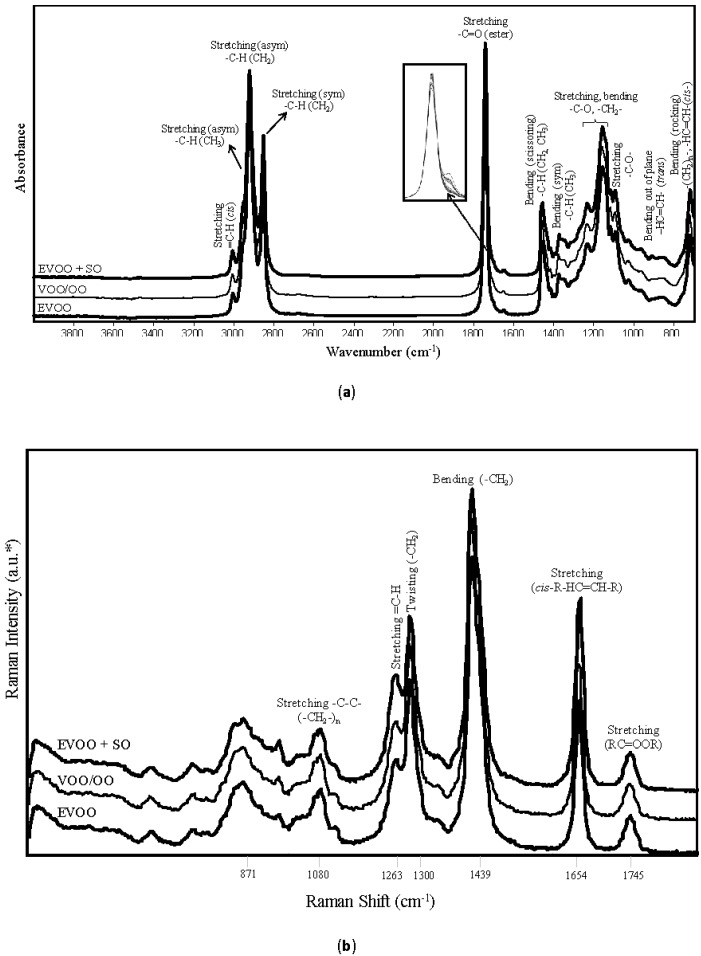
(**a**) FT-IR spectrum and band assignments of different quality olive oils at frequency of 4000–700 cm^−1^ collected using a portable 5-reflections ZnSe crystal ATR system equipped with a temperature-controlled accessory. (**b**) Raman spectrum of different quality olive oils at frequencies of 200–1850 cm^−1^ collected using a compact benchtop Raman system working with 1064 nm excitation laser. EVOO: Extra virgin olive oil, VOO/OO: Blend of virgin olive oil and olive oil, EVOO + SO: Extra virgin olive oil + Sunflower oil. *a.u.: Arbitrary units.

The characteristic FT-IR absorption spectra of different grades of olive oil samples and their corresponding band assignments for specific functional groups are displayed in Figure 1a. Visual inspection of the spectra showed close resemblance in their spectral profiles throughout the mid-IR region (4000–700 cm^−1^) (Figure 1a), similar to those previously reported by Rohman and others (2017) [54]. Key absorbance signals included the band at 3010 cm^−1^ associated with =C–H stretching of cis olefins, the 2900–2800 cm^−1^ range related to–C–H symmetrical and asymmetrical stretching vibrations (CH_2_ and CH_3_), the band centered at 1746 cm^−1^ associated to the stretching vibrations of the ester carbonyl (–C=O) functional group of triglycerides, and the band at 1465 cm^−1^ associated with C–H bending (scissoring) vibration of the CH_2_ group. The band at 1377 cm^−1^ corresponds to the C–H bending (symmetrical) vibration of the CH_3_ group, and the shoulder band centered at 1417 cm^−1^ due to the rocking vibrations of the C-H bonds of cis-disubstituted olefins. Finally, the fingerprint region from 1200 to 1000 cm^−1^ represented the unique stretching and bending vibrations of –C–O and –CH_2_– vibrational modes. Overall, important spectral regions for revealing possible EVOO adulteration included the band intensities at 3010–2800 cm^−1^ related to the triglyceride fatty acid composition and level of unsaturation of the oils, and the relative proportion between the triglyceride ester-linkage (COOR) band at 1742 cm^−1^ and the C=O absorption of FFAs at 1711 cm^−1^. An increase in the band intensity at 1711 cm^−1^ correlates with the increase in FFA content of oil [55].

The Raman spectra for selected olive oil samples and their band assignments for specific functional groups are given in Figure 1b. The band at 1080 cm^−1^ was associated with C-C stretching vibration (-CH_2_-)*_n_*, while the band at 1263 cm^−1^ was associated with =C-H in-plane deformation of a conjugated cis double bond (cis-R-HC=CH-R) and related with monounsaturated fatty acids. The band at 1300 cm^−1^ was related to -C-H twisting motion (-CH_2_), and the band at 1439 cm^−1^ was associated with -C-H bending (-CH_2_) modes. The band at 1654 cm^−1^ was related to C=C stretching (cis-R-HC=CH-R) from polyunsaturated fatty acids. The band at 1745 cm^−1^ was associated with C=O stretching vibration (RC=OOR) [9,56]. Different pure olive oils (EVOO, VOO, OO) did not show major differences throughout the measured Raman spectrum (Figure 1b), but olive oil adulterated with other vegetable oils displayed marked differences (higher bands) in the band intensities at 1263 and 1654 cm^−1^. As mentioned earlier, those bands correspond to monounsaturated and polyunsaturated fatty acids, and an increase in their band intensities has been related to an increasing weight percentage of unsaturated fatty acids in olive oils [9,56].

### 3.3. Pattern Recognition Modeling Using FT-IR and Raman Spectroscopy

The FT-IR and Raman spectral data were analyzed using soft independent modeling of class analogy (SIMCA) for the authentication of EVOO and detection of adulteration, either by blending with other vegetable oils or replacing of EVOO with lower olive oil grades, such as refined, pomace, or lampante olive oils. Single-class and multi-class pattern recognition strategies were assessed either by using a binary (authentic EVOO vs. VOO/OO blends and EVOO adulterated with vegetable oils) or multiple (authentic EVOO, VOO/OO blends and EVOO adulterated with vegetable oils) class approach based on the information provided by the Aydin Commodity Exchange Laboratories and California Olive Oil Council, along with our reference tests’ results.

**Figure 2:**
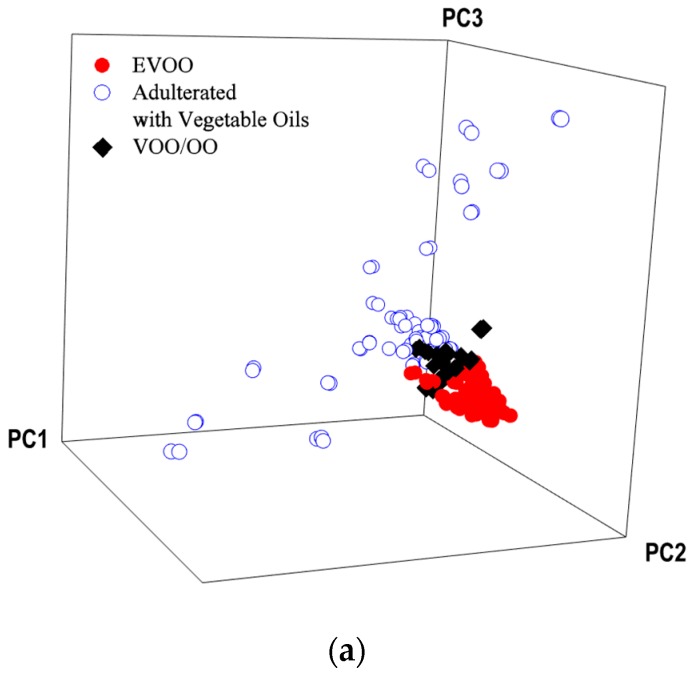

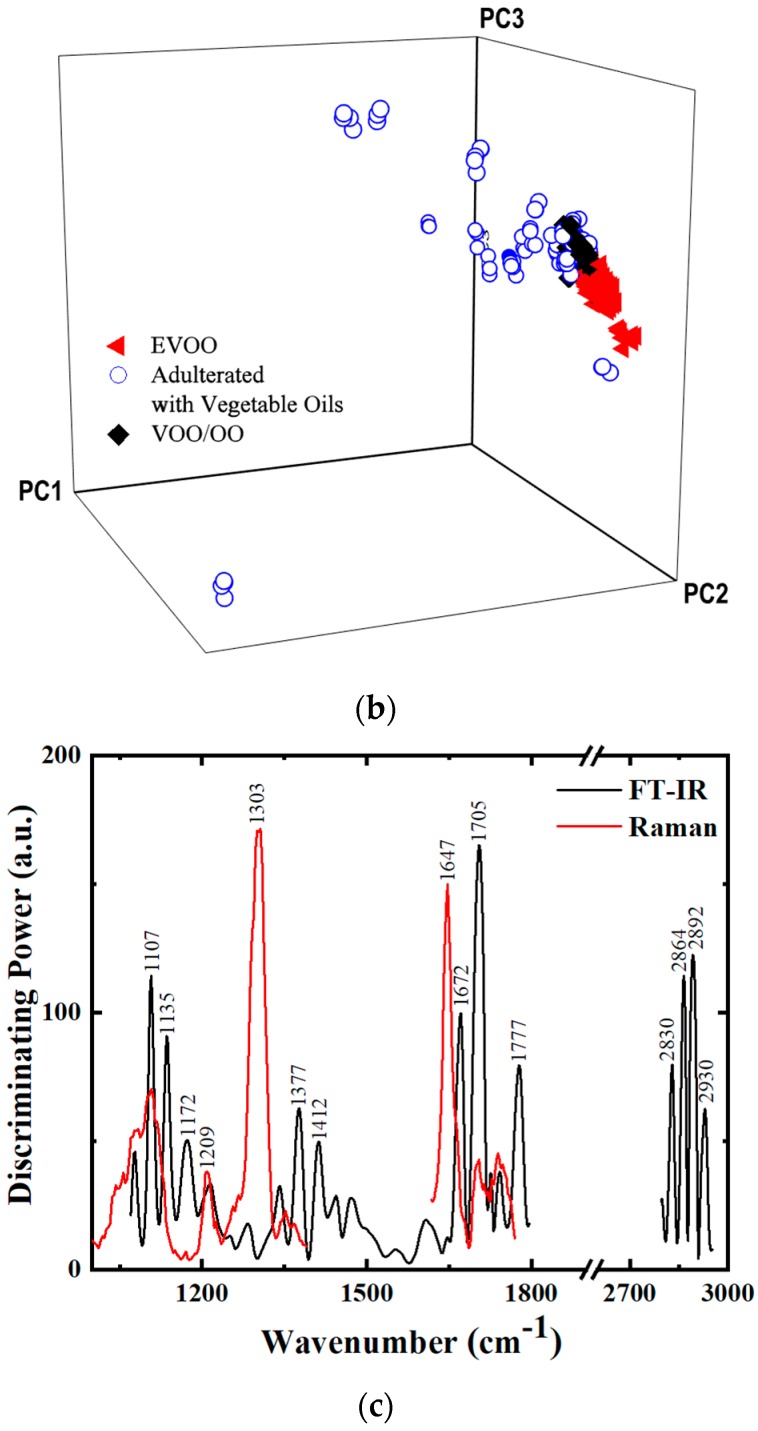
(**a**) Soft independent modeling of class analogy (SIMCA) 3D projection plots of spectral data for olive oil samples collected by (**a**) portable FT-IR and (**b**) compact benchtop Raman spectrometers. EVOO: Extra virgin olive oil, VOO/OO: Blend of virgin olive oil and olive oil. (**c**) SIMCA discriminating plot based on the mid-infrared and Raman spectra of olive oils using an FT-IR and a Raman spectrometer, showing bands and regions responsible for class separation.

A multi-class approach was implemented for the FT-IR spectral data that comprised three different groups including EVOO, VOO/OO blends, and adulterated olive oil with vegetable oils. The class projection plot (Figure 2a) showed compact clusters for the EVOO and VOO/OO blends, indicating similar chemical composition among samples in their class, while the marked compositional differences in EVOO adulterated with different vegetable oils were reflected by the large spread of samples in the class projection plot. A SIMCA parameter that correlated to the chemical differences between classes was the interclass distances (ICD) and gave values ranging from 2.6 (EVOO & VOO/OO blends) to 6.1 (VOO/OO blends & EVOO with other vegetable oils) (Table 2). In the SIMCA models, two different classes with an ICD >3 are considered significantly different from each other [36]. Overall, all classes were largely independent of one another, requiring three to five PCs to explain 99% of the variance within groups and the cross-validation showed zero misclassifications, which indicates that the model should be robust and minimizes over-fitting. The SIMCA discriminating power plot (Figure 2c) showed that the clustering of different olive oil grades and adulteration were explained by the bands centered at 2920 and 2850 cm^−1^, corresponding to CH_2_ asymmetric and symmetric stretching vibrations, and 1742, 1711, and 1098 cm^−1^,which correspond to the stretching vibrations of the carbonyl bonds (–C=O) in acylglycerides, and the 1670 cm^−1^ band, related to the olefinic trans C=C stretching vibrations.

The predictive performance of the multi-class calibration model was determined by using an independent validation set that included fifteen EVOOs, five VOO/OO blends, and nine EVOOs adulterated with other vegetable oils. By including the information of additional classes (i.e., VOO/OO blends and EVOO with other vegetable oils), the sensitivity and specificity of the SIMCA models were 100% for all the oil classes (Table 3). Since authentication studies are often approached as a one-class classification analysis, the adulterants are usually unknown [57]. A one-class SIMCA model was developed for EVOO based on the infrared spectra of genuine samples, and any adulterated samples were classified as outliers when tested against the PCA model boundaries. The performance of the calibration models was evaluated by using an independent validation set that consisted of 15 authentic EVOO and 74 non-authentic (VOO/OO and EVOO with other vegetable oils) samples. All EVOO samples were correctly predicted (TP = 15 and FN = 0) as belonging to its target class, resulting in 100% sensitivity, indicating that the one-class model was capable of accurately identifying authentic EVOO samples. On the other hand, eight of the non-authentic samples were predicted as EVOO (FP = 8, TN = 66), resulting in 89% specificity (Table 3), revealing that the model had adequate ability to detect adulterated samples. The one-class model correctly predicted all EVOO mixed with cheaper vegetable oils, while eight out of twenty-seven VOO/OO were predicted as belonging to the EVOO class.

A similar approach was taken for the Raman spectral data collected from the oils to detect EVOO adulteration. The class projection plot is given in Figure 2b. The multi-class SIMCA model gave ICDs ranging from 0.9 to 7.0, with the largest dissimilarity of spectral features obtained between authentic EVOO and its mixtures with other vegetable oils (ICD = 7.0), while the ICD differentiating EVOO from VOO and its blends with refined olive oils was 0.9 (Table 4). Wold and Sjöström (1977) described that distances between class models larger than one indicate real differences, and if two models are not independent, the interclass distance is close to zero [58]. The classes required three to five PCs to explain 98% of the variance within groups, and the cross-validation showed zero misclassifications. The SIMCA discriminating power plot (Figure 2c) was dominated by the bands centered at 1652 and 1306 cm^−1^, associated with the alkene νC=C stretch and in-phase methylene twisting vibrations, respectively. The minor bands at 920 and 856 cm^−1^ were attributed with bending vibrations of trans (C=C) and stretching vibrations of methylene chain skeleton, respectively [8]. An independent validation set was used to evaluate the predictive performance of the SIMCA models. Sensitivity evaluated the capability of our classification model to identify EVOO, while specificity determined the ability of our model to discriminate the adulterated or mislabeled samples. The sensitivity and specificity values for the single and multi-class models for Raman spectroscopy are given in Table 3. The multi-class model gave 100% sensitivity and specificity, which means that models generated by Raman spectra could effectively detect authentic EVOO samples from adulterated oils with excellent accuracy. Although the ICD separating the pure EVOO from VOO and its blends with refined olive oils was 0.9, the model gave perfect predictions. SIMCA single class models developed from Raman models correctly predicted all authentic EVOO (TP = 15 and FN = 0; 100% sensitivity). However, out of the 74 validation samples that were either mislabeled (lower olive oil grades) or adulterated with other vegetable oils, the one-class model failed to identify 25 samples that were predicted as pure EVOO (FP = 25, TN = 49; sensitivity = 66%). A total of 12 VOO/OO blends and 13 adulterated samples were classified as EVOO.

Similar to our findings, Li et al. (2018), Philippidis et al. (2017), and Zhang et al. (2011) were also be able to differentiate olive oils from vegetable oils including waste cooking oil, sunflower, rapeseed, soybean, corn, and canola oil by using Raman spectroscopy [8,9,56]. However, we report for the first time the discrimination of EVOO from their different grades (VOO and OO). Our data showed the challenges in detecting EVOO from OO, as very few unique compounds, monochloropropanediol esters, and glycidyl esters formed in the refining process can be used as markers for authentication [59]. By including the additional features from the class assigned to VOO and OO samples to the supervised model allowed to improve the discriminability of the classifiers providing the best accuracy for authentication of EVOO without false positives. Furthermore, EVOO adulterated with pomace olive oil showed marked FT-IR and Raman spectral differences allowing straightforward detection by pattern recognition analysis.

### 3.4. Development of PLSR Models Using FT-IR and Raman Spectroscopy

**Figure 3:**
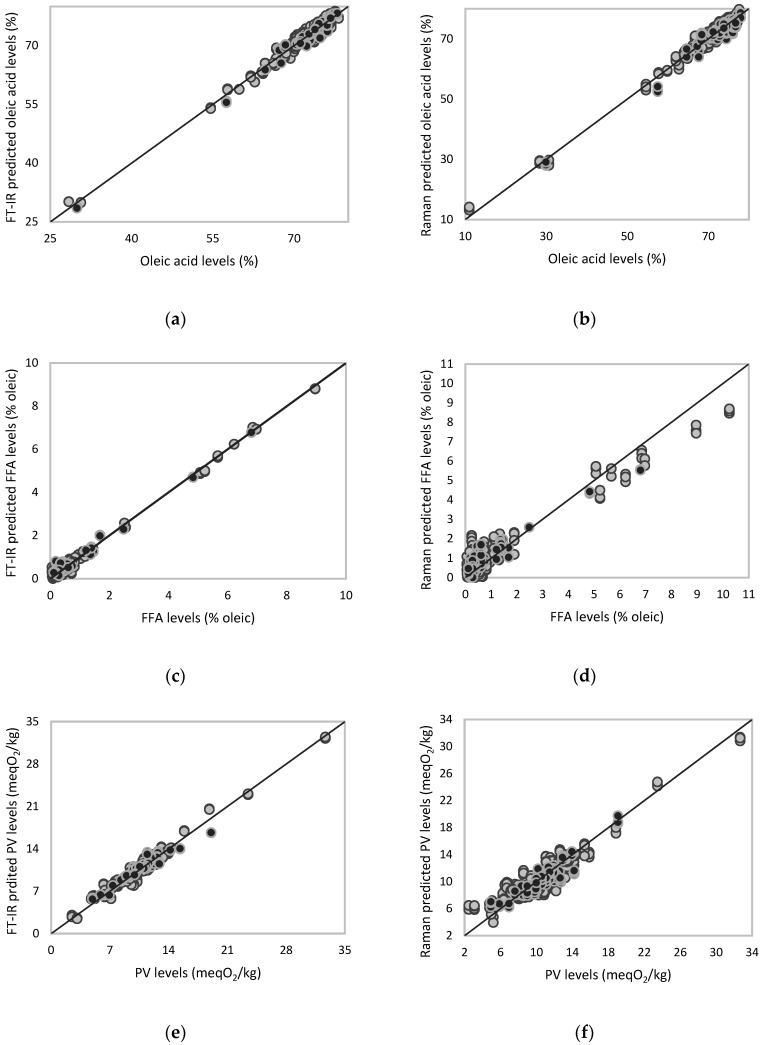
Partial least squares regression (PLSR) calibration and external validation plots for oleic (**a** and **b**), free fatty acids (**c** and **d**), and peroxide value (**e** and **f**) levels in olive oil samples using a portable 5-reflections FT-IR and compact benchtop Raman instrument, respectively. Grey circles represent samples in calibration set; black circles represent samples in external validation set.

Extra virgin olive oil (EVOO) quality and its freshness degrade over time due to its high level of monounsaturated fatty acid content (oleic acid). Therefore, it is important to monitor the main quality parameters (FFA, PV, PPP, TPC, and major fatty acid content) in EVOO throughout the olive oil production process and during the storage. Taking this into account, the FT-IR and the Raman spectra collected using the portable and compact benchtop units were employed to develop quantitative models with partial least squares regression (PLSR) based on reference values for free fatty acids (FFA), peroxide value (PV), pyropheophytin (PPP), total polar compounds (TPC), and major fatty acids (palmitic, stearic, oleic, linoleic, and linolenic) (Figure 3). Samples were randomly divided into two groups as calibration and external validation sets, eighty percent of the total number of samples were randomly chosen to generate the calibration set and the other twenty percent were used to generate the external validation set to assess the robustness of the models. The performance statistics of each model, the minimum and maximum values, and the number of samples used in each calibration and external validation set were given in Table 5. If a sample has high leverage and/or residual, it was identified as an outlier and excluded from the model, therefore the total number of samples in each model could be different from each other. For the best model performances, and to eliminate the irrelevant, noisy, and unreliable variables (wavenumbers), specific wavenumbers were selected from the FT-IR and Raman spectral regions for each analyte. Depending on the quality parameter, cross-validation (leave-one-out) identified three to six factors to generate the FT-IR and Raman calibration models.

Table 5 shows the performance statistics for the PLSR calibration and external validation models that were obtained for five major fatty acids (palmitic, stearic, oleic, linoleic, and linolenic) tested in olive oils and the main indices (FFA, PV, PPP, and TPC) that monitor olive oil quality. The SECV values for each calibration model was similar to the standard error of prediction (SEP) of their corresponding external validation model (Table 5), demonstrating the robustness of the generated models. The SEP values ranged from 0.01% to 1.5% for the five major fatty acids present in the tested olive oils. Our models showed superior performance statistics for the estimation of fatty acid profiles (lower correlation coefficient and SEP) than those reported by Gurdeniz and others (2010) for extra virgin olive oils using a benchtop FT-IR unit [60]. Furthermore, our calibration and validation models for the major fatty acids had similar performances to those reported by [61], but they employed 13–14 factors to acquire those statistics, which probably over-fitted the models. Using the same FT-IR and Raman spectral data, we also generated models for the main olive oil quality indices including FFA, PV, PPP and TPC and their performance statistics are given in Table 5. Overall, the FT-IR regression models gave superior performance than those generated by Raman spectroscopy. For example, the model generated by FT-IR for estimation of FFA levels gave correlation coefficient of validation (R_v_) of 1.00 and standard error of prediction (SEP) of 0.23% by using three factors, while the Raman model gave an R_v_ of 0.93 and SEP of 0.55 by using six factors (Table 5). Gouvinhas and others (2015) obtained good performances (R^2^ = 0.99) on the prediction of FFA content in EVOO at different maturation stages by using a shorter excitation wavelength laser (488 nm) over the spectral range of 950–1800 cm^−1^ [62].

## 4. Conclusions

The present study was designed to evaluate portable FT-IR and compact benchtop Raman technology for the nondestructive authentication of premium EVOO and detect adulteration with the addition of lower grades of olive oils or other vegetable oils. Multi–class pattern recognition algorithms defining EVOO, VOO/OO (lower quality olive oils), and adulterated EVOO with vegetable oils classes allowed accurate classification with perfect sensitivity and specificity. However, a single-class approach resulted in diminished sensitivity, resulting in the misclassification of VOO and OO samples as EVOO. Our data demonstrated the importance of developing supervised classification models, including relevant a priori knowledge in the training set, especially samples with similar compositional make-up, such as lower quality olive oils, to develop reliable methods to reveal EVOO fraud. Furthermore, the same spectra were used to generate multivariate regression models to predict major quality parameters, including levels of fatty acids, %FFA, PV, PPP, and TPC. Both the portable FT-IR and compact benchtop 1064 nm Raman were promising technologies for “in-situ”, non-destructive, simple and quick identification of possible adulteration of EVOOs. However, the portable FT-IR unit gave the best classification and quantitation results, even when comparing against reported SEP collected in benchtop systems. Our approach showed sensitivity and specificity to detect EVOO fraud, even with lower processing grade olive oils, and provides rapid quantitative analysis for monitoring oil quality parameters.

## Figures and Tables

**Table 1:** Reference concentration levels for the compounds measured in olive oil samples.

		EVOO ^a^	VOO/OO ^b^	Mixture ^c^
Palmitic (%)	Range	9.8–17.4	10.6–18.1	5.3–18.9
	Mean	13.2	13.4	12.1
	SD	1.7	1.9	2.8
Stearic (%)	Range	2.7–2.9	2.7–3.1	2.7–3.5
	Mean	2.8	2.8	2.9
	SD	0	0.1	0.2
Oleic (%)	Range	62.0–78.2	57.7–76.5	11.0–76.9
	Mean	72.6	71.5	66.9
	SD	3.8	4.4	14
Linoleic (%)	Range	4.5–14.8	6.0–17.7	5.6–76.0
	Mean	8.5	9.5	15.1
	SD	2.2	2.4	14
Linolenic (%)	Range	0.6–0.8	0.7–0.9	0.1–5.8
	Mean	0.7	0.7	1
	SD	0	0.1	0.9
Free Fatty Acid (%)	Range	0.1–0.7	0.1–1.9	0.1–10.3
	Mean	0.4	0.5	2.1
	SD	0.2	0.5	2.7
Peroxide Value (meqO_2_/kg)	Range	4.8–13.7	3.1–13.2	2.5–32.7
	Mean	9.8	10	11.7
	SD	2	2.5	4.9
Pyropheophytin (%)	Range	7.0–14.9	5.6–20.6	12.5–25.5
	Mean	11.5	13.2	19.8
	SD	2.3	3	3
Total Polar Compound (%)	Range	2.5–8.5	4.0–9.8	5.5–17.8
	Mean	5.2	6.6	8.7
	SD	1.1	1.5	2.4

^a^ EVOO: Extra virgin olive oil, ^b^ VOO/OO: Blend of virgin olive oil and olive oil, ^c^ Mixture: Adulterated olive oil with vegetable oils (corn, sunflower, soybean, and canola oil).

**Table 2:** Interclass distances between three classes of olive oils based on the SIMCA class projections for the FT-IR spectra collected in the 700–4000 cm^−1^ region.

Groups	EVOO ^a^	VOO/OO Blends ^b^	EVOO with other Vegetable Oils ^c^
EVOO	0		
VOO/OO blends	2.6	0	
EVOO with other vegetable oils	5.2	6.1	0

^a^ EVOO: Extra virgin olive oil, ^b^ VOO/OO: Blend of virgin olive oil and olive oil, ^c^ Adulterated EVOO with other vegetable oils (corn, sunflower, soybean, and canola oil).

**Table 3:** Sensitivity and specificity values of SIMCA multi- and single-class models obtained from FT-IR and Raman spectroscopy.

Model Types		Samples	Sensitivity (%)	Specificity (%)
Multi-Class	FT-IR	VOO/OO blends ^b^	100	100
		EVOO ^a^ with other vegetable oils	100	100
	Raman	VOO/OO blends	100	100
		EVOO with other vegetable oils ^c^	100	100
One-Class	FT-IR		100	89
	Raman		100	66

^a^ EVOO: Extra virgin olive oil, ^b^ VOO/OO: Blend of virgin olive oil and olive oil, ^c^ Adulterated EVOO with other vegetable oils (corn, sunflower, soybean, and canola oil).

**Table 4:** Interclass distances between three classes of olive oils based on the SIMCA class projections for the Raman spectra collected in the 250–1850 cm^−1^ region.

Groups	EVOO ^a^	VOO/OO Blends ^b^	EVOO with other Vegetable Oils ^c^
EVOO	0		
VOO/OO blends	0.9	0	
EVOO with other vegetable oils	7.0	5.9	0

^a^ EVOO: Extra virgin olive oil, ^b^ VOO/OO: Blend of virgin olive oil and olive oil, ^c^ Adulterated EVOO with other vegetable oils (corn, sunflower, soybean, and canola oil).

**Table 5:** Performance statistics of calibration and external validation models developed by using portable FT-IR and compact benchtop Raman spectroscopy.

Technique	Parameter	Calibration Model					External Validation Model			
		Range	*N* ^a^	Factor	SECV ^b^	Rcal	Range	*N* ^c^	SEP ^d^	Rval
FT-IR	Palmitic (%)	5.3–18.9	120	6	0.44	0.98	6.5–18.1	30	0.53	0.98
	Stearic (%)	2.7–3.6	120	4	0.03	0.98	2.7–3.5	30	0.02	0.99
	Oleic (%)	11.0–78.2	120	4	1.13	0.99	29.9–78.0	30	1.41	0.99
	Linoleic (%)	4.5–76.0	120	4	1	0.99	5.7–41.0	30	1.4	0.98
	Linolenic (%)	0.5–1.8	117	4	0.02	0.99	0.6–1.0	29	0.02	0.97
	FFA (%)	0.1–10.3	118	3	0.17	1	0.1–6.8	30	0.23	0.99
	PV (meqO_2_/kg)	2.5–32.7	120	5	0.65	0.98	4.9–19.1	30	0.79	0.96
	Pyropheophytin (%)	5.6–25.5	87	6	1.47	0.96	10.7–23.5	22	1.46	0.94
	TPC (%)	2.5–17.8	120	6	0.54	0.97	3.3–13.3	30	0.59	0.97
Raman	Palmitic (%)	5.3–18.9	120	6	0.84	0.91	6.5–18.1	30	0.99	0.92
	Stearic (%)	2.7–3.6	120	5	0.04	0.96	2.7–3.5	30	0.04	0.97
	Oleic (%)	11.0–78.2	120	6	1.33	0.99	29.9–78.0	30	1.78	0.98
	Linoleic (%)	4.5–76.0	120	4	1.09	0.99	5.7–41.0	30	1.63	0.99
	Linolenic (%)	0.5–1.8	118	6	0.02	0.99	0.6–1.0	30	0.01	0.98
	FFA (%)	0.1–10.3	118	6	0.55	0.94	0.1–6.8	30	0.52	0.93
	PV (meqO_2_/kg)	2.5–32.7	120	4	1.31	0.92	4.9–19.1	30	1.11	0.92
	Pyropheophytin (%)	7.0–25.5	85	5	1.93	0.92	10.7–20.5	21	1.55	0.92
	TPC (%)	2.5–17.8	119	6	0.76	0.94	3.3–13.3	30	0.83	0.93

^a^ Number of samples used in calibration models. ^b^ Standard error of cross validation. ^c^ Number of samples used in external validation models. ^d^ Standard error of prediction.

## References

[1] Gelpí E., Posada de la Paz M., Terracini B., Abaitua I., Gómez de la Cámara A., Kilbourne E.M., Lahoz C., Nemery B., Philen R.M., Soldevilla L. (2002). The spanish toxic oil syndrome 20 years after its onset: A multidisciplinary review of scientific knowledge. Environ. Health Perspect..

[2] Bajoub A., Bendini A., Fernández–Gutiérrez A., Carrasco–Pancorbo A. (2018). Olive oil authentication: A comparative analysis of regulatory frameworks with especial emphasis on quality and authenticity indices, and recent analytical techniques developed for their assessment. A. review. Crit. Rev. Food Sci. Nutr..

[3] United States International Trade Commission (2013). Olive Oil: Conditions of Competition between U.S. and Major Foreign Supplier Industries.

[4] Aparicio R., Morales M.T., Aparicio–Ruiz R., Tena N., García–González D.L. (2013). Authenticity of olive oil: Mapping and comparing official methods and promising alternatives. Food Res. Int..

[5] Milanez K.D.T.M., Nóbrega T.C.A., Nascimento D.S., Insausti M., Band B.S.F., Pontes M.J.C. (2017). Multivariate modeling for detecting adulteration of extra virgin olive oil with soybean oil using fluorescence and UV–Vis spectroscopies: A preliminary approach. LWT-Food Sci. Technol..

[6] Aroca–Santos R., Cancilla J.C., Pérez–Pérez A., Moral A., Torrecilla J.S. (2016). Quantifying binary and ternary mixtures of monovarietal extra virgin olive oils with UV–vis absorption and chemometrics. Sens. Actuators B Chem..

[7] Durán Merás I., Domínguez Manzano J., Airado Rodríguez D., Muñoz de la Peña A. (2018). Detection and quantification of extra virgin olive oil adulteration by means of autofluorescence excitation–emission profiles combined with multi–way classification. Talanta.

[8] Li Y., Fang T., Zhu S., Huang F., Chen Z., Wang Y. (2018). Detection of olive oil adulteration with waste cooking oil via Raman spectroscopy combined with iPLS and SiPLS, Spectrochim. Acta-Part A Mol. Biomol. Spectrosc..

[9] Philippidis A., Poulakis E., Papadaki A., Velegrakis M. (2017). Comparative Study using Raman and Visible Spectroscopy of Cretan Extra Virgin Olive Oil Adulteration with Sunflower Oil. Anal. Lett..

[10] Mendes T.O., da Rocha R.A., Porto B.L.S., de Oliveira M.A.L., dos Anjos V.D.C., Bell M.J. (2015). Quantification of Extra–virgin Olive Oil Adulteration with Soybean Oil: A Comparative Study of NIR, MIR, and Raman Spectroscopy Associated with Chemometric Approaches. Food Anal. Methods.

[11] Rohman A., Man Y.B.C. (2010). Fourier transform infrared (FTIR) spectroscopy for analysis of extra virgin olive oil adulterated with palm oil. Food Res. Int..

[12] Di Girolamo F., Masotti A., Lante I., Scapaticci M., Calvano C.D., Zambonin C., Muraca M., Putignani L. (2015). A simple and effective mass spectrometric approach to identify the adulteration of the mediterranean diet component extra–virgin olive oil with corn oil. Int. J. Mol. Sci..

[13] Calvano C.D., De Ceglie C., D’Accolti L., Zambonin C.G. (2012). MALDI–TOF mass spectrometry detection of extra–virgin olive oil adulteration with hazelnut oil by analysis of phospholipids using an ionic liquid as matrix and extraction solvent. Food Chem..

[14] Lorenzo I.M., Pavón J.L.P., Laespada M.E.F., Pinto C.G., Cordero B.M. (2002). Detection of adulterants in olive oil by headspace–mass spectrometry. J. Chromatogr. A.

[15] Šmejkalová D., Piccolo A. (2010). High–power gradient diffusion NMR spectroscopy for the rapid assessment of extra–virgin olive oil adulteration. Food Chem..

[16] Xu Z., Morris R.H., Bencsik M., Newton M.I. (2014). Detection of virgin olive oil adulteration using low field unilateral NMR. Sensors.

[17] Vigli G., Philippidis A., Spyros A., Dais P. (2003). Classification of edible oils by employing 31P and 1H NMR spectroscopy in combination with multivariate statistical analysis. A proposal for the detection of seed oil adulteration in virgin olive oils. J. Agric. Food Chem..

[18] Fragaki G., Spyros A., Siragakis G., Salivaras E., Dais P. (2005). Detection of extra virgin olive oil adulteration with lampante olive oil and refined olive oil using nuclear magnetic resonance spectroscopy and multivariate statistical analysis. J. Agric. Food Chem..

[19] Dugo G., Rotondo A., Mallamace D., Cicero N., Salvo A., Rotondo E., Corsaro C., Mannina L., Salvo A. (2015). Enhanced detection of aldehydes in extra–virgin olive oil by means of band selective NMR spectroscopy. Physica A.

[20] Rotondo A., Mannina L., Salvo A. (2019). Multiple Assignment Recovered Analysis (MARA) NMR for a Direct Food Labeling: The Case Study of Olive Oils. Food Anal. Methods..

[21] Kumar S., Kahlon T., Chaudhary S. (2011). A rapid screening for adulterants in olive oil using DNA barcodes. Food Chem..

[22] Mildner–Szkudlarz S., Jeleń H.H. (2010). Detection of olive oil adulteration with rapeseed and sunflower oils using mos electronic nose and smpe–ms. J. Food Qual..

[23] Knolhoff A.M., Croley T.R. (2016). Non–targeted screening approaches for contaminants and adulterants in food using liquid chromatography hyphenated to high resolution mass spectrometry. J. Chromatogr. A.

[24] Tengstrand E., Rosén J., Hellenäs K.-E., Åberg K.M. (2013). A concept study on non–targeted screening for chemical contaminants in food using liquid chromatography–mass spectrometry in combination with a metabolomics approach. Anal. Bioanal. Chem..

[25] Coates J. A Review of New Small–Scale Technologies for Near Infrared Measurements. https://www.americanpharmaceuticalreview.com/Featured-Articles/163573-A-Review-of-New-Small-Scale-Technologies-for-Near-Infrared-Measurements/.

[26] Ellis D.I., Muhamadali H., Haughey S.A., Elliott C.T., Goodacre R. (2015). Point–and–shoot: Rapid quantitative detection methods for on–site food fraud analysis–moving out of the laboratory and into the food supply chain. Anal. Methods..

[27] Mossoba M.M., Azizian H., Fardin–Kia A.R., Karunathilaka S.R., Kramer J.K.G. (2017). First Application of Newly Developed FT–NIR Spectroscopic Methodology to Predict Authenticity of Extra Virgin Olive Oil Retail Products in the USA. Lipids.

[28] Hirri A., Gammouh M., Gorfti A., Kzaiber F., Bassbasi M., Souhassou S., Balouki A., Oussama A. (2015). The use of Fourier transform mid infrared (FT–MIR) spectroscopy for detection and estimation of extra virgin olive oil adulteration with old olive oil. Sky J. Food Sci..

[29] Pan M., Sun S., Zhou Q., Chen J. (2018). A Simple and Portable Screening Method for Adulterated Olive Oils Using the Hand–Held FTIR Spectrometer and Chemometrics Tools. J. Food Sci..

[30] Jiménez-Carvelo A.M., Osorio M.T., Koidis A., González-Casado A., Cuadros-Rodríguez L. (2017). Chemometric classification and quantification of olive oil in blends with any edible vegetable oils using FTIR–ATR and Raman spectroscopy. LWT-Food Sci. Technol..

[31] Georgouli K., Martinez Del Rincon J., Koidis A. (2017). Continuous statistical modelling for rapid detection of adulteration of extra virgin olive oil using mid infrared and Raman spectroscopic data. Food Chem..

[32] Firestone D. (2012). Method Cd 8-53: Peroxide Value-Acetic Acid-Chloroform Method. Official Methods and Recommended Practices of the American Oil Chemists’ Society.

[33] (2004). European Pharmacopoeia, European Pharmacopoeia 01/2005:20501, in: Counc. Eur., vol. 1. https://kampoeng2013.files.wordpress.com/2015/12/european-pharmacopoeia-5-with-all-supplements.pdf.

[34] (2016). ISO 29841:2009/AMD 1:2016, Vegetable Fats and Oils—Determination of the Degradation Products of Chlorophylls a and a’ (Pheophytins a, a’ and Pyropheophytins)—Amendment 1. https://www.iso.org/standard/66396.html.

[35] Berrueta L.A., Alonso–Salces R.M., Héberger K. (2007). Supervised pattern recognition in food analysis. J. Chromatogr. A.

[36] Kvalheim O.M., Karstand T.V., Brereton R.G. (1992). SIMCA—Classification by Means of Disjoint Cross Validated Principal Components Models. Multivariate Pattern Recognition in Chemometrics: Illustrated by Case Studies.

[37] Checa–Moreno R., Manzano E., Capitán–Vallvey L.F. (2014). Characterisation and classification of binders used in art materials at the class and the subclass level. Anal. Methods..

[38] Abdi H. (2010). Partial least squares regression and projection on latent structure regression (PLS Regression). Wiley Interdiscip. Rev. Comput. Stat..

[39] Romía M.B., Bernàrdez M.A., Sun D.-W. (2009). Multivariate Calibration for Quantitative Analysis. Infrared Spectroscopy Food Qulity Analytical Control.

[40] Feng S., He A., Wang D., Kang B. (2019). Diagnostic significance of miR–210 as a potential tumor biomarker of human cancer detection: An updated pooled analysis of 30 articles. Onco Targets Ther..

[41] USDA United States Standards for Grades of Olive Oil and Olive–Pomace Oil; 2010; pp. 9–10. https://www.ams.usda.gov/sites/default/files/media/Olive_Oil_and_Olive–Pomace_Oil_Standard%5B1%5D.pdf.

[42] International Olive Council (2006). Trade Standards Applying to Olive Oils and Olive Pomace Oils.

[43] Boudour–Benrachou N., Plard J., Pinatel C., Artaud J., Dupuy N. (2017). Fatty Acid Compositions of Olive Oils from Six Cultivars from East and South–Western Algeria. Adv. Food Technol. Nutr. Sci. Open J..

[44] Cicero N., Albergamo A., Salvo A., Bua G.D., Bartolomeo G., Mangano V., Rotondo A., Di Stefano V., Di Bella G., Dugo G. (2018). Chemical characterization of a variety of cold–pressed gourmet oils available on the Brazilian market. Food Res. Int..

[45] Klikarová J., Rotondo A., Cacciola F., Česlová L., Dugo P., Mondello L., Rigano F. (2019). The Phenolic Fraction of Italian Extra Virgin Olive Oils: Elucidation Through Combined Liquid Chromatography and NMR Approaches. Food Anal. Methods..

[46] Van Doosselaere P., Hamm W., Hamilton R.J., Calliauw G. (2013). Production of Oils. Edible Oil Process.

[47] Choe E., Min D.B. (2007). Chemistry of deep–fat frying oils. J. Food Sci..

[48] (1991). Commission Regulation (EEC) No 2568/91. https://eur-lex.europa.eu/legal-content/EN/TXT/PDF/?uri=CELEX:01991R2568-20151016&from=EN.

[49] Casal S., Malheiro R., Sendas A., Oliveira B.P.P., Pereira J.A. (2010). Olive oil stability under deep–frying conditions. Food Chem. Toxicol..

[50] Vossen P., Aparicio R., Harwood J. (2013). Growing Olives for Oil. Handbook Olive Oil Analysis Properties.

[51] Guillaume C., Gertz C., Ravetti L. (2014). Pyropheophytin a and 1,2–diacyl–glycerols over time under different storage conditions in natural olive oils. JAOCS J. Am. Oil Chem. Soc..

[52] Frankel E.N., Mailer R.J., Schoemaker C.F., Wang S.C., Flynn J.D. (2010). Tests Indicate that Imported “Extra Virgin” Olive Oil often Fails International and USDA Standards. https://www.kretareserve.com/info/oliveoilappendix071510.pdf.

[53] Osawa C.C., Gonçalves L.A.G., Gumerato H.F., Mendes F.M. (2012). Study of the effectiveness of quick tests based on physical properties for the evaluation of used frying oil. Food Control.

[54] Rohman A., bin Che Man Y., Ismail A., Hashim P. (2017). FTIR spectroscopy coupled with chemometrics of multivariate calibration and discriminant analysis for authentication of extra virgin olive oil. Int. J. Food Prop..

[55] Bendini A., Cerretani L., Di Virgilio F., Belloni P., Bonoli–Carbognin M., Lercker G. (2007). Preliminary evaluation of the application of the ftir spectroscopy to control the geographic origin and quality of virgin olive oils. J. Food Qual..

[56] Zhang X.F., Zou M.Q., Qi X.H., Liu F., Zhang C., Yin F. (2011). Quantitative detection of adulterated olive oil by Raman spectroscopy and chemometrics. J. Raman Spectrosc..

[57] Zhang L., Li P., Sun X., Mao J., Ma F., Ding X., Zhang Q. (2015). One–class classification based authentication of peanut oils by fatty acid profiles. RSC Adv..

[58] Wold S., Sjostrom M., Kowalski B.R. (1977). SIMCA: A method for analyzing chemical data in terms of similarity and analogy. Chemometrics Theory and Application, American Chemical Society Symposium Series 52.

[59] Yan J., Oey S.B., van Leeuwen S.P.J., van Ruth S.M. (2018). Discrimination of processing grades of olive oil and other vegetable oils by monochloropropanediol esters and glycidyl esters. Food Chem..

[60] Gurdeniz G., Ozen B., Tokatli F. (2010). Comparison of fatty acid profiles and mid–infrared spectral data for classification of olive oils. Eur. J. Lipid Sci. Technol..

[61] Maggio R.M., Kaufman T.S., Del Carlo M., Cerretani L., Bendini A., Cichelli A., Compagnone D. (2009). Monitoring of fatty acid composition in virgin olive oil by Fourier transformed infrared spectroscopy coupled with partial least squares. Food Chem..

[62] Gouvinhas I., Machado N., Carvalho T., De Almeida J.M.M.M., Barros A.I.R.N.A. (2015). Short wavelength Raman spectroscopy applied to the discrimination and characterization of three cultivars of extra virgin olive oils in different maturation stages. Talanta.

